# Veterinary Association of Manitoba

**Published:** 1896-11

**Authors:** W. A. Dunbar

**Affiliations:** Secretary


### SOCIETY PROCEEDINGS.

### VETERINARY ASSOCIATION OF MANITOBA.

THE semi-annual meeting was held in the city of Brandon on August 29th. The members present were: Drs. Swenerton, of Wawanesa, Vice-President; Dunbar, of Winnipeg, Secretary-Treasurer; Rutherford, Portage la Prairie; McLoughrey, Moosomin; Coote, Winnedosa; Ward, Oak Lake; Torrance, Fisher, and Coxe, Brandon. The President, Dr. Young, of Manitou, being unavoidably absent, Vice-President Swenerton occupied the chair.

Dr. Rutherford, in an appropriate speech, introduced the subject of typhoid-malarial fever in the horse, a disease of a somewhat fatal character which has been prevalent in the Red River Valley for several years, being specially noticeable during the months of July and August. An interesting and profitable discussion on the cause, nature, and treatment of the disease followed, which was participated in by Drs. Torrance, Fisher, Swenerton, Dunbar, and others.

Influenza and pernicious anæmia, two diseases very common among the horses of this province, received from the members present the consideration which their importance demand.

Dr. Rutherford described, in a manner which reflected great credit on his faculty of observation, the ante-mortem and post-mortem symptoms of pernicious or progressive anæmia, called in India "surra."

The meetings of the Association are steadily gaining in interest and are doing a good work in advancing the veterinary art in Manitoba, and every member who desires to assist in elevating the standard of the profession will find it much to his advantage to be present, if possible, at every future meeting.

The next annual meeting will be held in the city of Winnipeg, in February, 1897.

W. A. DUNBAR,
Secretary.

